# Non-tuberculous cutaneous mycobacterioses^☆^^☆☆^

**DOI:** 10.1016/j.abd.2021.04.005

**Published:** 2021-07-16

**Authors:** Lais Bastos Nogueira, Carina Nogueira Garcia, Marcela Santos Corrêa da Costa, Monica Brauner de Moraes, Patrícia Shu Kurizky, Ciro Martins Gomes

**Affiliations:** a Hospital Universitário de Brasília, Universidade de Brasília, Brasília, DF, Brazil; b Postgraduate Program in Tropical Medicine, Universidade de Brasília, Brasília, DF, Brazil; c Hospital da Criança de Brasília José Alencar, Brasília, DF, Brazil; d Postgraduate Program in Medical Sciences, Universidade de Brasília, Brasília, DF, Brazil

**Keywords:** Culture media, Diagnosis, Molecular biology, Mycobacterium infections, nontuberculous

## Abstract

Non-tuberculous mycobacteriosis, previously known as atypical, anonymous, opportunistic, or unclassified mycobacteriosis, refers to pathogenic mycobacterioses other than those caused by *Mycobacterium tuberculosis* and *Mycobacterium leprae*. These mycobacteria are known for their environmental distribution, mainly in water and soil. The incidence of non-tuberculous mycobacteriosis has been increasing in all countries and skin infections are being increasingly studied, mainly with the increase in immunosuppressive conditions and the development of new medications that affect immunological function. In the present article, a detailed narrative review of the literature is carried out to study the main non-tuberculous mycobacteriosis that cause diseases of the skin and appendages. The article also aims to present a historical context, followed by epidemiological, microbiological, and clinical characteristics of these diseases. Practical considerations about the diagnosis and treatment of non-tuberculous mycobacteriosis are detailed.

## Introduction

Mycobacteria are pathogens of utmost importance for human health. The *Mycobacterium* genus consists of a group of aerobic actinobacteria that cause mainly tuberculosis and leprosy.^1^ Despite the reduced scientific and social attention given to the two main mycobacterioses, these are still the most well-known diseases in this class. Other types of mycobacteriosis, despite the increasing incidence, are scarcely studied. These diseases have a considerable impact on the quality of life of vulnerable populations and at-risk groups, such as the immunosuppressed.

Non-tuberculous mycobacteriosis (NTMs), formerly known as an atypical, anonymous, opportunistic, or unclassified mycobacteriosis, are pathogenic mycobacterioses that are not caused by *Mycobacterium tuberculosis* or *Mycobacterium leprae*.^2^, ^3^ NTMs are known for their ubiquitous environmental distribution, especially in water and soil.^1^ The pathogenic behavior of the *Mycobacterium* genus is extremely variable, with some representatives behaving as mandatory pathogens, others as opportunistic pathogens and others being considered non-pathogenic.^4^ There are also several animals that are colonized or infected with mycobacteria and that can act as reservoirs.^5^ In recent years, attention to the study of NTMs has been increasing, due to the better identification of these pathogens, the advent of more sensitive identification techniques, of immunosuppressive diseases and drugs.^4^ There has been a significant increase in the number of publications on the topic.^6^

This article reviews the NTMs responsible for several diseases of the skin and appendages. In the historical context, the main epidemiological, microbiological, and clinical aspects of these diseases are addressed, as well as the diagnosis and treatment of NTMs.

## History

The evolution of mycobacterioses is related to the history of Medicine and discoveries in the area of ​​microbiology. After Antonie van Leeuwenhoek's description of microorganisms and studies that led to the creation of Koch's postulates, there has been a great evolution in the knowledge about mycobacterial infections, but the totality of this interaction is far from being known.^7^

Mycobacteria are believed to have originated more than 150 million years ago.^8^ The epidemiology of *Mycobacterium ulcerans* infection is evidence of this history, as its presence in specific places in the African continent and South America indicates its existence before the division of the continents.^1^ Initially, the genus *Mycobacterium* only included the causative agents of tuberculosis and leprosy. In 1959, Ernest Runyon developed the classification of mycobacteria according to the generation of chromophores and the velocity of replication.^9^ The advent of molecular biology, with the amplification of nucleic acid developed by Kary Banks Mullis, allowed a detailed study of the genetic load of living organisms and the development of diagnostic methods that are more sensitive than direct examination, histopathological analysis or culture.

## Epidemiology

Data on the epidemiological behavior of NTMs are scarce due to their environmental characteristics and extreme neglect of the condition. NMT infections become more frequent each decade.^6^ This is partly due to the greater capacity for detecting these pathogens and partly due to the advent of new immunosuppressive diseases and medications. However, this higher frequency has been observed even in regions where pulmonary tuberculosis is decreasing, showing that this effect is not only a consequence of the greater capacity for detection.^10^

### Epidemiology of non-tuberculous cutaneous mycobacteriosis in South America

Data on the epidemiology of NTMs in South America and Brazil also do not allow precise conclusions about regional behavior. Local factors and similarities with other environments can act as important clues for prediction and control actions. Increasingly more frequent case reports have demonstrated that the main sources of NTM infections are the environment and hospital infections after surgical procedures.

Most of the southern part of the American continent is located in tropical and subtropical regions. This distribution favors the transmission of aquatic environment mycobacteria.^11^ Suspected cases of *M. ulcerans* infection in countries such as Peru and Brazil call attention to the environmental factors similar to endemic countries and will be detailed in subsequent sections.^12^ The economic development of this region can also be a factor for transmission that occurs during surgical or aesthetic procedures.^13^

### Taxonomy, microbiology and genetic characterization

Mycobacteria belong to the order *Actinomycetaceae* and to the family *Mycobacteriaceae*.^2^ The genus *Mycobacterium* is considered relatively homogeneous taxonomically, and its components have genotypic and phenotypic characteristics that can easily differentiate them from other genera.^3^ Mycobacteria are mandatory aerobic, acid-fast bacilli that have a mycolic acid structure.^1^

Mycobacteria are widely distributed in the environment, including water sources, animals and human beings. The genus consists of more than 170 species, most of which are of no clinical interest.^2^ Mycobacteria show a peculiar behavior in the laboratory analysis, which consists of slow growth and the need for complex culture media for growth.^2^ It is important to mention that *M. leprae* has not yet been grown on artificial media.^14^ This makes it difficult to study the natural characteristics of the pathogens, as well as to develop diagnostic tests.

In 1959, Ernest Runyon proposed a classification for NTMs associated with lung infections (Table 1).^9^ In this classification, still adopted at present, the growth rate of the mycobacteria and the production of chromogens are taken into consideration. As for growth, they are divided into slow-growing (>7 days) and fast-growing (<7 days) mycobacteria. It is important to emphasize that even fast-growing mycobacteria multiply much more slowly than other types of bacteria. When the production of chromogens is also considered, mycobacteria are divided into Type I - Photochromogenic: produce yellowish colonies when exposed to light; Type II - Scotochromogenic: regardless of exposure to light, they also produce yellowish colonies; Type III - Achromogenic: produce discreet or no pigmentation. Type IV is classified only because it also represents rapid growth. Depending on the pigmentation and genotypic characteristics, NTMs are classified by some authors into five groups: *Mycobacterium fortuitum*, *Mycobacterium chelonae-abscessus*, *Mycobacterium mucogenicum*, *Mycobacterium smegmatis,* and fast-growing and early pigmentation NTM groups.^15^ The phenotypic division, based on culture media, has no relation to the clinical behavior of mycobacterioses.

**Table 1:** Classification of non-tuberculous mycobacteria according to their growth rate and chromogenic characteristics. Runyon classification.

**Slow growing (>7 days)**
Type I - Photochromogenic
*M. kansasii, M. simiae, M. marinum, M. asiaticum*
Type II - Scotochromogenic
*M. scrofulaceum, M. gordonae, M. flavescens, M. szulgai*
Tipo III - Achromogenic
*M. avium complex*, *M. ulcerans, M. terrae, M. haemophilum*
**Fast-growing (<7 days)**
Type IV
*M. abscessus, M. chelonae, M. fortuitum*

### Pathogenesis and immune response to mycobacteria

The clinical expression of mycobacterioses depends on a complex interaction between the pathogen and the host from the initial infection to the most advanced stages of the disease. Initially, the mycobacteria involved play an important role in infectivity and pathogenicity. The mycobacterial antigens significantly modulate the immune response. As an example, one can mention molecules such as phenolic glycolipid I (PGLI), present in *M. leprae*, which is an important and specific immune escape factor. Strains of *Mycobacterium bovis* that expressed PGLI by genetic engineering demonstrated an increased capacity to interact with complement 3 receptor (CR3) and consequent greater evasion of the immune response by human macrophages and other proinflammatory responses.^16^ The amount of pathogens exposed to the immune system is also an important modulating factor of infection and pathogenicity. Studies performed with flow cytometry show that the greater the exposure to mycobacterial antigens, the greater the differentiation of T cells.^17^

Although immunity generated by T cell differentiation is the most widely studied response to mycobacterial infection, innate immunity, the initial immune barrier, is extremely protective.^18^ Macrophages, dendritic cells, neutrophils, and natural killer cells are the main cells involved in this type of response.^19^ Other cells are also of known importance in this process, such as epithelial and mast cells.^19^ At this stage, cell mechanisms such as phagocytosis, apoptosis and autophagy are used in the fight against infecting mycobacteria.^19^

Several signals and receptors play an important role in the innate immune response against mycobacteria. The identification of the pathogens is the first step in the fight against infection. Receptors such as toll-like receptors (TLRs), nod-like receptors (NLRs), c-type lectin receptors (CLRs), scavenger receptors (such as MSR1, MARCO, and CD36), and CD14, AIM2, and AhR.67–71 receptors are very important for the identification of the mycobacteria and the beginning of the phagocytosis process.^19^ Additionally, these receptors constitute the initial step for the production of pro-inflammatory cytokines and the beginning of the complex immune cascade. It is also interesting to note that the activation of inflammasomes is part of this immune cascade. This response acts as a trigger for the interleukin (IL)1β-mediated immune response.^19^

The adaptive response is the branch of immune response most frequently studied in chronic infections, such as those caused by mycobacteria. T-lymphocytes are essential for the control of mycobacterioses.^20^ In this branch, antigen-presenting cells, such as dendritic cells, activate and induce the proliferation of naive TCD4 + lymphocytes, which in turn initiate the phagocytosis process.^20^ Studies with mice also demonstrated that antigen-presenting cells migrate to the mediastinal lymphatic organs, ultimately resulting in the production of specific CD4+ T lymphocytes.^20^

The classic dichotomy between the effective cell immune response and the humoral immune response, also described as the T helper (Th)1 – Th2 balance, still explains much of the host's immune kinetics, although recent studies have shown that this is only one of the arms of the immune response. A minority of individuals who come into contact with mycobacteria will develop clinical disease. Those who develop the disease and Th1 immune response based on an effective cellular response will have localized infection, with an intense granulomatous response. At the other end of the spectrum, patients who, due to genetic or environmental reasons are unable to develop an effective cellular immune response, will have disseminated infections and mycobacteria-rich lesions.^21^ To this end, the humoral immune response is intense but ineffective for defense against pathogens. Leprosy is one of the most classic clinical examples of this balance between Th1–Th2 response.^14^ In part, this balance can also be expected for other mycobacterioses.

As an example, transplanted patients or patients with genetic diseases that alter the expression of interferon (IFN)-γ and IL-12 show a higher risk of infection by mycobacteria, demonstrating the importance of these cytokines in the pathogenesis of NTMs.^2^ The central role of IFN-γ, IL-12 and tumor necrosis factor (TNF)-α in the pathogenesis of NTM infections is well evidenced by the high incidence in children with disabilities in this axis, as well as in individuals who use TNF neutralizing agents, but not in patients using immunobiological inhibitors of IL-17 and IL-23. An important characteristic of the immune interaction between the host and mycobacteria is already well described in infections caused by *M. tuberculosis*. Mycobacteria can be latent for several years in healthy individuals.^20^ This implies the possibility of severe clinical conditions in immunosuppressed patients or those who receive immunosuppressive drugs. Prophylaxis protocols for latent tuberculosis have significantly reduced cases of reactivated tuberculosis. This latency and the chance of reactivation are not well defined or studied in NTMs.

### Transmission

The NTM form of contagion and transmission is still a subject of debate. Due to their environmental behavior, means of contagion such as trauma and contact with vectors are often supported in cases of skin infections. In general, human-to-human transmission, if any, is not considered a relevant source. There are no studies proving the possibility that the infection can be transmitted from one individual to another, or between animals. Molecular biology studies have shown that strains of *M. abscessus* that infect patients with cystic fibrosis can be inhaled from fomites or aerosols.^10^ Skin and soft tissue diseases are caused by trauma or surgical procedures.^10^ Recently, Drummond et al. reviewed several risk factors for NTM infection.^10^ In addition to contact with dry and aquatic environments that may contain mycobacteria, factors related to the hosts should also be observed. Immunosuppression related to genetic factors or with immunosuppressive drugs should always be considered as risk factors.

## Clinical aspects

### Clinical manifestations according to pathogenic agents

Mycobacteria that cause NTMs are considered bacteria with low pathogenic power. However, they can present different clinical forms, especially in immunosuppressed patients. Ninety percent of the diseases caused by NTMs result in chronic lung disease.^10^ The clinical manifestations are usually divided into four main clinical syndromes: pulmonary disease, skin and soft tissue disease, lymphadenitis, and disseminated disease.^10^ Other manifestations such as chronic otitis media, otomastoiditis, and musculoskeletal infections have also been described.

If mucocutaneous forms and the form of contagion that originated the lesion are taken into consideration, it is important to describe the following clinical manifestations: lymphadenitis, foreign body-type manifestations, infection related to a central venous catheter, and disseminated infection. These presentations may change over time or be associated.

### *Mycobacterium ulcerans* infection, a serious public health problem

*M. ulcerans* infection is the third most common mycobacteriosis worldwide and has a high impact on public health in developing countries, in addition to being considered a neglected disease.^11^ These factors justify special attention to this condition. The disease is also recognized by other names such as Buruli's ulcer, Bairnsdale ulcer, Tora ulcer, Searl disease, and Daintree disease. However, these names should be avoided.^11^

The disease is more prevalent in countries located on the west coast of Africa and in Australia, but countries located in South America and the western Pacific Ocean have already reported cases.^1^ There is a case recorded in Brazil, and the country's environmental characteristics are similar to those of countries that actively report infection by *M. ulcerans*.^12^ However, the endemicity of the disease has not been confirmed or reproduced, and it is worth remembering that the precise identification of mycobacterial species is not an easy task.

This mycobacteriosis mainly affects children who live in rural, humid areas, with swamps and flooded areas with slow-moving water sources. Some probable risk factors that have already been described include gardening, insect bites and proximity to lake regions. *M. ulcerans* is a slow-growing Runyon Group III mycobacterium. As an important characteristic, this mycobacterium produces the toxin called mycolactone, probably associated with the severe tissue destruction observed in cases with long duration.^1^

The initial lesions are characterized by papules or nodules, usually in exposed areas of the lower limbs. ^11^ The tissue necrosis progresses to an ulcer, relatively painless despite evident and visible tissue destruction. The lesion can remain relatively stable or progress to necrosis of the adjacent tissues and result in bone infiltration. On average, 60% of cases occur in the lower limbs; however, any other skin area may be affected. Despite the low mortality, more severe untreated cases can develop osteomyelitis and sequelae, with loss of limb function.

For small ulcers, surgical excision is the treatment of choice. The recommended treatment consists of the combination of antibiotics to prevent drug resistance, mainly rifampicin associated with clarithromycin or streptomycin for eight weeks.^22^ Quinolones can be used instead of clarithromycin (moxifloxacin or ciprofloxacin).^22^ If deep structures are involved, treatment can be prolonged. In cases of significant necrosis, surgical debridement, local heating, and other procedures may be necessary.

### *Mycobacterium avium* complex (MAC)

Diseases caused by the *M. avium complex* have been increasingly common with the advent and persistence of immunosuppressive conditions. The complex is formed by *Mycobacterium avium* and *Mycobacterium intracellulare*. These mycobacteria are among the most frequent causes of mycobacterioses in developed countries.^23^ Skin infections are caused by trauma.^24^ Several types of clinical presentation can be found, including plaques, pustules, panniculitis, lymphadenitis, among others.^23^ In immunosuppressed patients, a disseminated disease associated with nonspecific systemic symptoms such as fever, night sweats, weight loss, bone pain, hepatosplenomegaly, and lymphadenopathy can occur. In these cases, skin involvement is uncommon, but when present, it takes the form of papulopustular lesions and necrotic ulcers, especially in the lower limbs. Antimicrobial treatment for MAC infections consists of the association of clarithromycin or azithromycin with ethambutol and rifampicin or rifabutin, for a prolonged period of time, depending on the clinical presentation of the infection.

### *Mycobacterium marinum*

*Mycobacterium marinum* is a mycobacterium associated with the aquatic environment, including being the cause of apparent disease in animals such as ornamental fish.^25^ This mycobacterium has shown increasing relevance and frequency for skin and soft tissue infection. Despite being a group I mycobacterium, according to the Runyon classification, it can grow in less than seven days in ideal culture media. However, its genetic load is more similar to that of a slow-growing mycobacteria.^25^

*M. marinum* mainly affects the skin and mucous membranes, since growth is slow at temperatures above 30 °C, which is characteristic of more central areas of the body. Most of the cases present localized, papular-nodular skin lesions, and there may be dissemination in some cases. In immunosuppressed patients, it can cause the sporotrichoid or rapidly disseminated form, with the latter being extremely rare.^25^

The treatment can be monotherapy for approximately three months. For more serious infections, a combination of antibiotics such as clarithromycin, ethambutol, rifampicin, and sulfamethoxazole + trimethoprim is recommended. Minocycline and doxycycline are viable alternatives.^1^ The duration of therapy is variable, and treatment maintenance is recommended for one to two months after symptoms resolution. Surgical debridement may be necessary.

### *Mycobacterium fortuitum*

*M. fortuitum* is found in the soil and in aquatic biofilms in various geographic regions.^26^ It is a fast-growing mycobacteria, and a member of Runyon group IV. Outbreaks of hospital disease have been reported and include operative wound infections, post-injection abscesses, and infections after endoscopy with contaminated scopes. Some cases have been described after several procedures such as piercings, tattoos, mesotherapy, acupuncture, pedicures, breast implants, and CO_2_ laser.^1^ Characteristically, it manifests by the presence of multiple painful subcutaneous nodules, but it can also cause pulmonary disease (unilateral non-cavitating pneumonia). Disseminated disease can occur in immunocompromised patients.

A combination of antibiotics including macrolides, fluoroquinolones, doxycycline, sulfamethoxazole + trimethoprim for at least four months is recommended for the treatment, in addition to surgical excision when necessary.^1^ In the case of severe or disseminated disease, parenteral treatment with two to three sensitive antimicrobials should be started, followed by oral treatment for 6 to 12 months.

### *Mycobacterium abscessus* complex (MABC)

The *M. abscessus complex* consists of a group of mycobacteria classified as Runyon group IV. They are fast-growing mycobacteria with a high frequency of microbial resistance and cause a wide variety of skin lesions.^27^ Disseminated infections as well as infections in other organs have also been described. There are 3 subspecies: *M. abscessus subsp. abscessus, M. abscessus subsp. massiliense* and *M. abscessus subsp. bolletii.*^27^ Cases contracted after functional or cosmetic surgery, as well as mesotherapy, acupuncture, tattoos, and pedicure procedures can be found in the literature.^1^, ^13^

There is no consensus for the treatment of infections by *M. abscessus* and it should be based on the sensitivity of *in vitro* isolates. Antibiotic treatment consists of the combined use of macrolides (clarithromycin as the drug of choice), associated in the initial phase with a parenteral antibiotic (amikacin, cefoxitin, tigecycline or imipenem). Other options include the combination of clarithromycin with linezolid or clofazimine. Treatment duration of more than four to six months is recommended. Debridement and surgical excision may be necessary.^1^

### *Mycobacterium chelonae*

**Figure 1:**
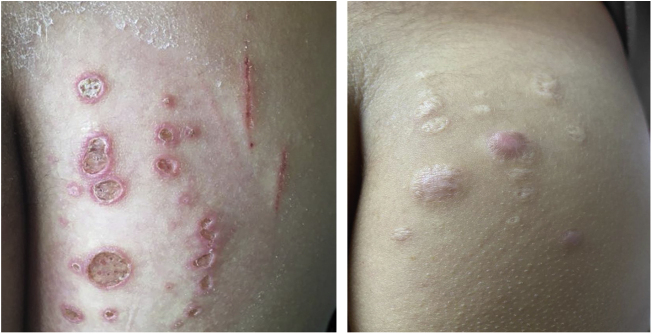
Non-tuberculous cutaneous mycobacteriosis caused by *Mycobacterium chelonae* in a child diagnosed by culture in Löwenstein-Jensen medium (identification by partial genetic sequencing of the rpoB gene). Treatment with monotherapy using clarithromycin resulted in complete cure.

This mycobacterium is found in aquatic environments, soil and surgical instruments.^28^ It belongs to the *M. fortuitum complex*, Runyon group IV.^28^ They are mycobacteria with low virulence that mainly affect immunosuppressed patients.^28^ Procedures such as tattoos, mesotherapy and acupuncture can be entry points for the infectious agent (Fig. 1). Combined treatments with macrolides, cefoxitin, imipenem, fluoroquinolones, and amikacin, in addition to the surgical approach are indicated.^1^ The clinical presentation is widely variable, with disseminated skin infection, showing erythematous nodules, with a tendency to drain and cause chronic ulcers. Cellulitis, localized abscesses or osteomyelitis, and central catheter-related infections may also occur.^15^

Combined treatments with macrolides, cefoxitin, imipenem, fluoroquinolones, linezolid, clofazimine, tobramycin, imipenem, and amikacin, in addition to the surgical approach, are indicated.^1^ Considering the observation of acquired resistance to clarithromycin, especially in adults, monotherapy should be avoided.^15^

### *Mycobacterium haemophilum*

*Mycobacterium haemophilum* is rarely associated with infections in humans; it most often occurs in Asia and is associated with severe comorbidities.^29^ There are cases acquired after cosmetic procedures. There is also an association with drug-induced immunosuppression or the use of TNF-blocking drugs.^1^ Treatment should be combined, including surgical approach and polychemotherapy with macrolides, fluoroquinolones, and rifampicin or rifabutin.^1^

### *Mycobacterium kansasii*

It is a slow-growing, photochromogenic mycobacterium classified as Runyon group I. It is usually described in immunosuppressed patients. It can cause infections in operative wounds with extension to deep tissues and bone.^30^ Treatment consists of a combination of isoniazid, rifampicin, ethambutol, and clarithromycin for 12 months.^1^

### BCG (bacillus Calmette-Guérin) vaccine disease

BCG (bacillus Calmette-Guérin) vaccine is made up of live, attenuated organisms (*Mycobacterium bovis*) and its main objective is to prevent severe forms (meningeal and miliary) of tuberculosis.^31^ Its administration is also recommended for household leprosy contacts as a way to prevent the disease.^32^ Some studies show that the BCG vaccine also provides some degree of protection against NTMs.^31^

In Brazil, a single dose of BCG is recommended, preferably within the first 12 hours after birth, while still in the maternity ward.^33^ In the case of household contact with a leprosy patient, the vaccination schedule should consider the vaccination history of the contact. If the contact is less than one year old and has already been vaccinated, there is no need for another dose of BCG. Those over one year of age, if they do not have a vaccination scar or have only one scar, should receive a dose of BCG. Those who received two doses or have two scars do not need an additional dose.^33^

After one to two weeks, a pustule is formed, with consequent ulceration. Between 6 and 12 weeks, a crust is formed, followed by a characteristic scar in approximately 95% of the vaccinees. Recurrence of the lesion may occur, even after its complete healing.

**Figure 2:**
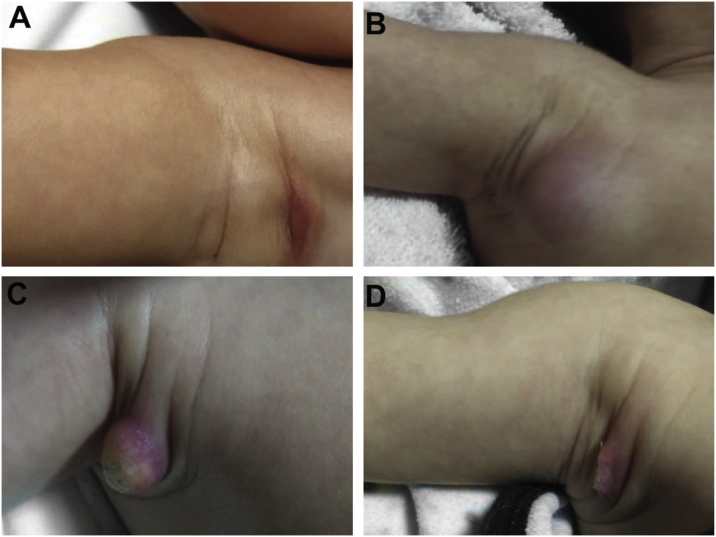
Adverse reaction to Bacillus Calmette-Guérin (BCG) vaccination. (A), Three months after the vaccination, showing regional axillary adenomegaly. (B), Four months after the vaccination with increased lymph node enlargement. (C), Five months after the vaccination with abscess formation. (D), Six months after the vaccination, with suppurative lesion.

During the normal course of the vaccine lesion, axillary, supra or infraclavicular axillary adenopathy without suppuration may occur, measuring up to 3 cm in diameter without general symptoms, with spontaneous resolution. However, local or systemic adverse effects can occur. These may be the result of the presence of immunodeficiency, the type of strain used, the application technique, and the amount of bacilli administered. Local and regional lesions are more frequent and respond better to treatment (Fig. 2).

Systemic dissemination or involvement rarely occurs. Lesions resulting from dissemination are those that go beyond the locoregional topography, which can affect the skin and distant lymph nodes, the osteoarticular system and viscera, developing tuberculosis-like lesions in the lungs, kidneys, and genitals. Generalized cases are represented by persistent fever, hepatomegaly, splenomegaly and multiple lymphadenitis. In these cases, the investigation of immunodeficiency is essential.

Adverse events should always be reported (Table 2). Most adverse effects of the BCG vaccine require clinical observation only. When the condition is local and persistent, with an ulcer measuring more than 1 cm, a cold abscess, and a difficult-to-heal granuloma, isoniazid 10 mg/kg/day (maximum dose of 400 mg) is indicated until complete lesion regression.^33^ In hot abscesses, the use of systemic antimicrobial agents for an acute, non-specific infectious skin process can be considered. In cases with lymphadenopathy > 3 cm, non-suppurated, it is not indicated to puncture, excise, or initiate isoniazid, and only follow-up is advised. When lymph node suppuration occurs, the prescription of isoniazid is indicated, at a dose of 10 mg/kg/day (maximum dose of 400 mg) until the regression of the condition is attained.^34^ In the lupoid reaction, a rare and late event, treatment with a triple regimen is recommended (isoniazid: 10 mg/kg/day, rifampicin: 10 mg/kg/day and ethambutol: 25 mg/kg/day), for two months, followed by isoniazid at a dose of 10 mg/kg/day and rifampicin: 10 mg/kg/day for four months.^34^

**Table 2:** Conduct in the presence of adverse effects after application of the Calmette-Guérin bacillus vaccine (BCG).

Adverse Event	Conduct
Local and Regional	
Ulcer > 1 cm	Clinical observation
	In the absence of cure: isoniazid (10 mg/kg/day, maximum dose of 400 mg) until complete regression. Follow-up for up to three months after treatment
Cold subcutaneous abscesses	Clinical observation
	Isoniazid (10 mg/kg/day, maximum dose of 400 mg) until complete regression. Follow-up for up to three months after treatment
Hot subcutaneous abscesses	Clinical observation
	Consider the presence of secondary infection and treat with antibiotic therapy for pyodermitis
Granulomas	Clinical observation
	In the absence of cure: isoniazid (10 mg/kg/day, maximum dose of 400 mg) until complete regression. Follow-up for up to three months after treatment
Nonsuppurative regional lymphadenopathy	Clinical observation
Suppurative regional lymphadenopathy	Clinical observation
	Isoniazid (10 mg/kg/day, maximum dose 400 mg) until significant reduction. Follow-up for up to three months after treatment
Keloid	Clinical observation
Lupoid reaction	Clinical observation
	Triple regimen with: isoniazid: 10 mg/kg/day; rifampicin: 10 mg/kg/day; ethambutol: 25 mg/kg/day for 2 months, followed by: isoniazid: 10 mg/kg/day and rifampicin: 10 mg/kg/day for four months
Disseminated	
Skin	Clinical observation
	Triple regimen with: isoniazid: 10 mg/kg/day; rifampicin: 10 mg/kg/day; ethambutol: 25 mg/kg/day for 2 months, followed by: isoniazid: 10 mg/kg/day and rifampicin: 10 mg/kg/day for four months
Osteoarticular	Clinical observation
	Triple regimen with: isoniazid: 10 mg/kg/day; rifampicin: 10 mg/kg/day; ethambutol: 25 mg/kg/day for 2 months, followed by: isoniazid: 10 mg/kg/day and rifampicin: 10 mg/kg/day for four months
Lymph nodes and single-organ involvement	Clinical observation
	Triple regimen with: isoniazid: 10 mg/kg/day; rifampicin: 10 mg/kg/day; ethambutol: 25 mg/kg/day for 2 months, followed by: isoniazid: 10 mg/kg/day and rifampicin: 10 mg/kg/day for four months
Generalized lesions affecting more than one organ	Clinical observation
	Triple regimen with: isoniazid: 10 mg/kg/day; rifampicin: 10 mg/kg/day; ethambutol: 25 mg/kg/day for two months, followed by: isoniazid: 10 mg/kg/day and rifampicin: 10 mg/kg/day for at least four months.

Source: *Ministério da Saúde Secretaria de Vigilância em Saúde Departamento de Vigilância das Doenças Transmissíveis B. Manual de Vigilância Epidemiológica de Eventos Adversos Pós-Vacinação*, 2020 URL http://editora.saude.gov.br.

In cases of cutaneous, osteoarticular, lymph node or visceral dissemination, the same treatment used for the lupoid reaction is indicated, in addition to biopsy, blood culture, and/or myeloculture and immunological evaluation of the patient.^34^

## Diagnosis

Laboratory diagnosis of NTMs depends on appropriate conditions for sample collection, storage and processing. As these bacteria are present in the environment, sample contamination can be frequent. False-positive results can lead to unnecessary treatments.

The longer the time between sample collection, transport and processing, the greater the chance of contaminant growth in culture media. The use of antibiotics with a proven mycobactericidal action, such as macrolides and quinolones, can lead to negative test results.^35^ Antibiotic therapy should be discontinued at least 15 days before the material is collected.

Polymerase Chain Reaction (PCR) testing is the best diagnostic method. Mycobactericidal drugs should always be avoided before collecting material for PCR. It is important to emphasize that tap water, or even filtered water, can lead to contamination of the material to be analyzed. The samples must be transported in a leak-proof container, sealed, certified and, ideally, without transportation media. If the shipment of the material takes more than one hour, it must be kept refrigerated at 4 °C.

### Direct examination and histopathological analysis

According to Griffith et al., the most recommended staining method for the observation of NTMs is fluorochrome staining.^35^ Ziehl-Neelsen or Kinyoun methods are also important, but less sensitive. Some pathogens, such as fast-growing NTMs, may be sensitive to the discoloration process. Immunohistochemistry still needs further studies for routine use in NTM identification.^36^

**Figure 3:**
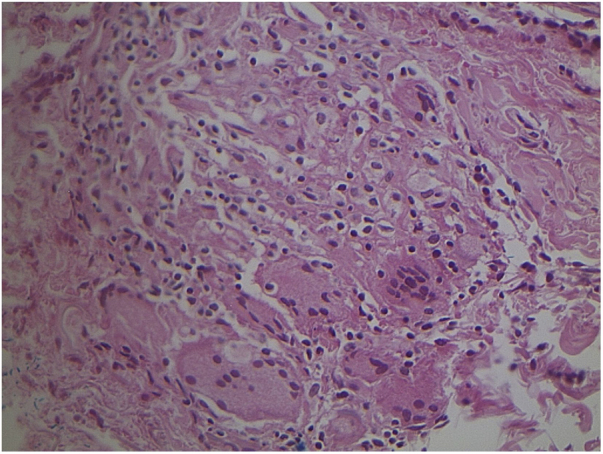
Non-specific granulomatous reaction caused by *Mycobacterium fortuitum* in an immunocompetent patient (Hematoxylin & eosin, ×40). Diagnosis was performed by amplification and partial genetic sequencing of the rpoB gene. Detection of mycobacteria can be hindered by the extensive granulomatous reaction.

**Figure 4:**
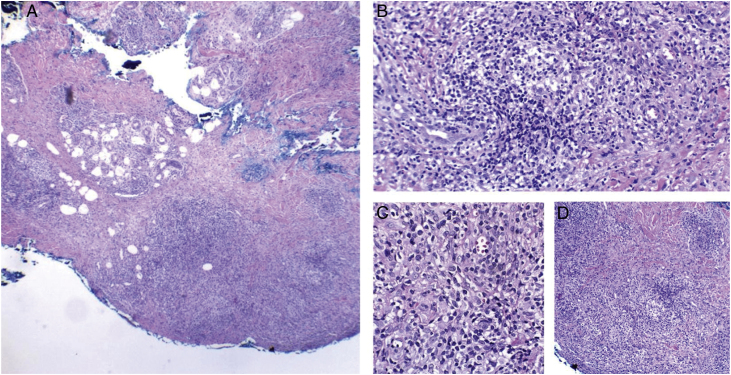
Cutaneous mycobacteriosis caused by *Mycobacterium haemophilum* in an immunosuppressed patient caused by the use of infliximab for 15 years. Intense infiltration, predominantly constituted of histiocytes, can be seen at the dermo-hypodermic junction, permeated by lymphocytes and neutrophils, but without forming halos, nodules, suppurative foci. Staining: Hematoxylin & eosin; diagnosis carried out by amplification and partial genetic sequencing of the folP1 gene. (A), Panniculitis, ×4; (B), Poorly organized granuloma, ×20; (C), Detail of lymphohistiocytic infiltrate, ×40; (D), Panniculitis with nodular outline, ×10.

Studies of tuberculosis cases that have less than 1000 bacilli/mL have, on average, a 10% chance of being positive on direct examination. Culture is a much more sensitive method.^37^ Recently, Li et al. reviewed the histopathological characteristics of 13 confirmed cases of cutaneous involvement by NTMs. In immunocompetent cases, pseudoepitheliomatous hyperplasia, intraepithelial abscesses, transepidermal elimination, and granulomas with necrosis and suppuration have been observed, but in immunosuppressed patients, granulomas, when present, aren’t well developed. ^6^ (Figure 3, Figure 4) It must be noted that these characteristics are similar to what is seen in other diseases found in our environment, such as paracoccidioidomycosis, leishmaniasis, sporotrichosis, and chromomycosis; however, the detection of mycobacteria in the skin is more difficult than in fungal diseases.

### Culture

Culture of skin fragments or body fluids is still considered one of the most important tests for the diagnosis and study of mycobacteria. The use of swabs should be avoided. The specificity is not 100%, as the growth of contaminating mycobacteria may occur. Liquid and solid media should be employed for this purpose.^38^ Recent advances have been described, but efforts to make this method a faster and simpler one should never be abandoned. Due to the long time required for the cultivation of NTMs, procedures that eliminate contamination by other bacteria and fungi must always be observed.^39^ The skin is considered to be a possibly contaminated sample and before seeding the samples, some laboratories recommend decontamination.^39^

The combination of liquid media, which allow faster and more solid growth, and solid media which allow the growth of rarer mycobacteria, should be used for the diagnosis of mycobacteriosis. Solid egg-based media, the classic Löwenstein-Jensen medium or its modified form can be used. Agar-based media include Middlebrook 7H10, Middlebrook 7H11, Mitchison 7H10, and 7H11 selective agar media and thin-layer agar culture (Table 3).^39^ Another important advantage of culture media is the possibility of testing antimicrobial resistance, which is extremely necessary considering the different types of mycobacteria that may be involved.

**Table 3:** Clinical aspects, diagnosis, and suggestion of treatment for adults with non-tuberculous mycobacteriosis according to the isolated species.

Clinical Aspects	Culture^a^	Treatment^b^
***Mycobacterium marinum***		
Infection after contact with aquatic environments. Most cases have localized, papular-nodular lesions in the extremities, and there may be dissemination in immunosuppressed individuals.	Slow growing (Photochromogenic)	For limited infections, monotherapy with clarithromycin (500–1000 mg/day), doxycycline (100 mg/day), minocycline (100 mg 2× a day), and sulfamethoxazole 400 mg + trimethoprim 80 mg 2× a day can be used for at least three months.
		For severe infections, rifampicin (450–600 mg/day) and ethambutol (15 mg/kg/day) can be used.
***Mycobacterium ulcerans***		
There is no confirmation of persistent sources of transmission in Brazil. Initial lesions appear as papules or nodules, progressing to relatively painless ulcers, despite evident and visible tissue destruction.	Slow growing (Achromogenic)	Combination of rifampicin (450–600 mg/day) and another active antibiotic such as clarithromycin (500–1000 mg/day), streptomycin (15 mg/kg/day) or quinolones for eight weeks (see current local or World Health Organization guidelines).
		Surgical intervention.
***Mycobacterium kansasii***		
Immunosuppressed patients. Operative wounds, including the involvement of deep tissues and bone.	Slow growing (Photochromogenic)	Traditional antibiotics at the doses used against tuberculosis has shown to be effective. Rifampicin (450–600 g/day), isoniazid (300 mg/day), ethambutol (15 mg/kg/day).
***Mycobacterium haemophilum***		
It rarely affects humans. It occurs after surgical procedures and in immunosuppressed patients.	Slow growing (Achromogenic)	Polychemotherapy containing clarithromycin (500–1000 mg/day), ciprofloxacin (500 mg 2× daily), and rifabutin (150–300 mg/day) are suggested.
***Mycobacterium fortuitum, Mycobacterium abscessus, Mycobacterium chelonae***		
Papules, nodules, plaques and abscesses. Disseminated infections can occur, especially in immunosuppressed individuals.	Fast-growing	For localized infections, polychemotherapy with clarithromycin (500–1000 mg/day), azithromycin (250–500 mg/day), ciprofloxacin (500 mg 2× a day), levofloxacin (500 mg/day), doxycycline (100 mg/day), minocycline (100 mg 2× a day) or sulfamethoxazole 400 mg + trimethoprim 80 mg 2× a day is recommended for severe infections. Initial parenteral treatment followed by oral treatment for six months to one year may be necessary with polychemotherapy.

a The combination of liquid media, which allows faster and more solid growth, also allowing the growth of rarer mycobacteria, which should be used for the diagnosis of mycobacteriosis. The precise identification of mycobacteria can be achieved using molecular biology strategies.

b Monotherapy should be avoided to prevent the appearance of bacterial resistance. Treatment should follow a sensitivity test for mycobacteria whenever possible.

### Methods using molecular biology

Molecular biology tests have revolutionized the study and diagnosis of mycobacterial infections. PCR transforms a scarce amount of DNA or RNA into a detectable amount. Several technical variations can be applied for diagnostic purposes.

**Figure 5:**
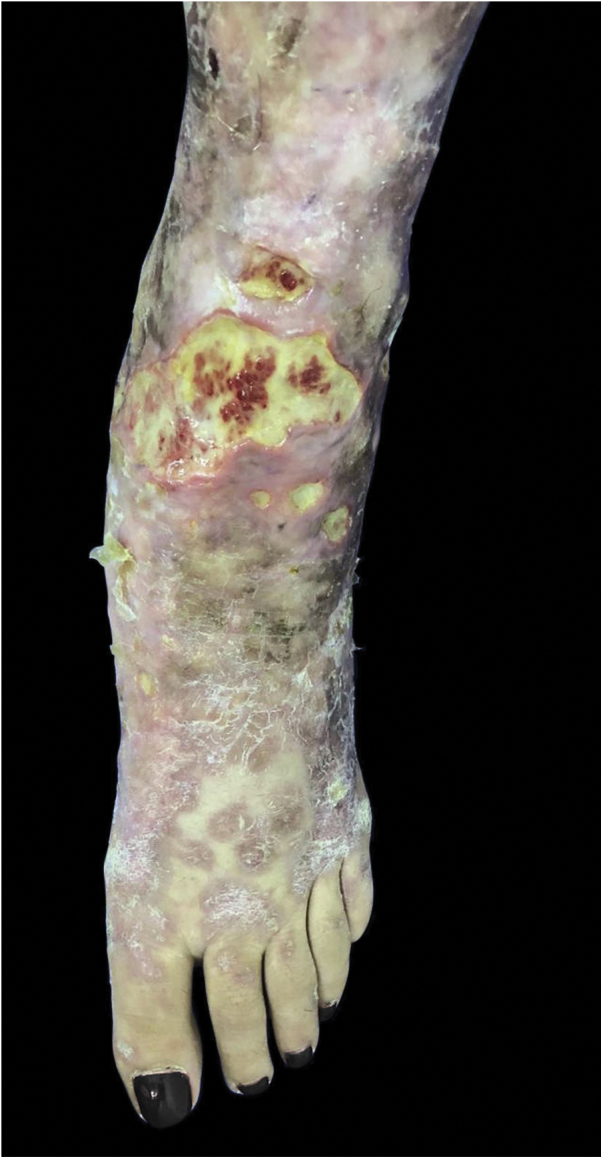
Non-tuberculous cutaneous mycobacteriosis. Polymerase chain reaction was positive for *Mycobacterium sp*. and negative for *Mycobacterium tuberculosis* specific primers. The genetic sequencing was inconclusive. The condition showed complete cure after treatment using a regimen for pulmonary tuberculosis associated with clarithromycin.

**Figure 6:**
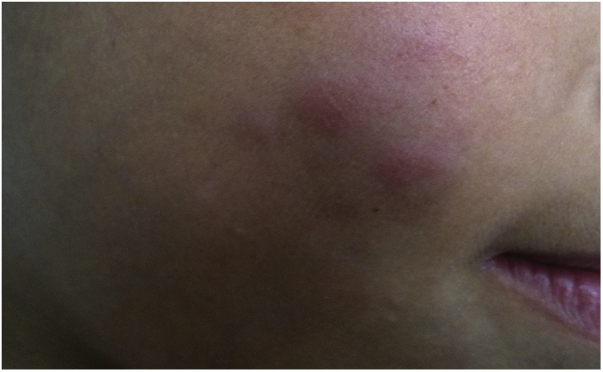
Cutaneous mycobacteriosis by *Mycobacterium fortuitum*, similar to lupus vulgaris. Diagnosis was performed by amplification and partial genetic sequencing of the rpoB gene.

Despite the range of available complementary exams, it is important to note that the sensitivity of these strategies is not 100%. For this reason, direct identification of the infeccious agent is not always possible, especially in places that do not have highly complex tests available. Effective antibiotic therapy prevents the detection of mycobacterial RNA but does not fully degrade the DNA. In the presence of a compatible clinical and histopathological examination and after excluding the differential diagnoses, an empirical therapeutic decision may be necessary (Figure 5, Figure 6).

## Treatment

Drugs and general recommendations are presented in the approach to the different mycobacterioses (Table 3).

### Vaccination

Studies have been developed aiming to produce vaccines against mycobacterioses. Tuberculosis prevention is the target of almost all of these attempts.^31^ Specific and effective vaccines against NTMs are not yet available.

BCG is a relatively low-cost vaccine, recommended in countries where tuberculosis is endemic.^33^ Currently, the vaccine is also used in public health strategies as prophylaxis to prevent the illness among household contacts in leprosy index cases, usually in patients who have never received BCG or who have received only one dose of this vaccine. Its protective effect against other mycobacteria has been the subject of debate and remains uncertain. However, its effect on endemic conditions such as *M. ulcerans* infection has been considered.^31^ The vaccine is contraindicated for immunosuppressed patients because it contains a living organism.

## Conclusion

The increasing incidence of NTMs justifies the further study of these conditions not only in adult individuals but also in children. The dermatologist has an important role in the identification and treatment of these diseases since the skin and its appendages can be affected, including after surgical and aesthetic procedures.

## Financial support

None declared.

## Authors' contributions

Lais Bastos Nogueira: Conceptualization, creation, editing and finalization.

Carina Nogueira Garcia: Conceptualization, creation, editing and finalization.

Marcela Santos Corrêa da Costa: Creation, editing and finalization.

Monica Brauner de Moraes: Creation, editing and finalization.

Patrícia Shu Kurizky: Conceptualization, creation, editing and finalization.

Ciro Martins Gomes: Conceptualization, creation, editing, review, finalization, coordination, supervision.

## Conflicts of interest

None declared.
